# Conservation and diversity of the eukaryotic SAGA coactivator complex across kingdoms

**DOI:** 10.1186/s13072-021-00402-x

**Published:** 2021-06-10

**Authors:** Ying-Jiun C. Chen, Sharon Y. R. Dent

**Affiliations:** 1 Department of Epigenetics and Molecular Carcinogenesis, The University of Texas MD Anderson Cancer Center, Smithville, TX USA; 2 The Center for Cancer Epigenetics, The University of Texas MD Anderson Cancer Center, Houston, TX USA

**Keywords:** SAGA complex, Transcription, Coactivator, Epigenetics, GCN5, Development, Stress, Disease, Cancer, Evolution

## Abstract

The SAGA complex is an evolutionarily conserved transcriptional coactivator that regulates gene expression through its histone acetyltransferase and deubiquitylase activities, recognition of specific histone modifications, and interactions with transcription factors. Multiple lines of evidence indicate the existence of distinct variants of SAGA among organisms as well as within a species, permitting diverse functions to dynamically regulate cellular pathways. Our co-expression analysis of genes encoding human SAGA components showed enrichment in reproductive organs, brain tissues and the skeletal muscle, which corresponds to their established roles in developmental programs, emerging roles in neurodegenerative diseases, and understudied functions in specific cell types. SAGA subunits modulate growth, development and response to various stresses from yeast to plants and metazoans. In metazoans, SAGA further participates in the regulation of differentiation and maturation of both innate and adaptive immune cells, and is associated with initiation and progression of diseases including a broad range of cancers. The evolutionary conservation of SAGA highlights its indispensable role in eukaryotic life, thus deciphering the mechanisms of action of SAGA is key to understanding fundamental biological processes throughout evolution. To illuminate the diversity and conservation of this essential complex, here we discuss variations in composition, essentiality and co-expression of component genes, and its prominent functions across Fungi, Plantae and Animalia kingdoms.

**Supplementary Information:**

The online version contains supplementary material available at 10.1186/s13072-021-00402-x.

## Background

The groundbreaking characterization of general control nonrepressed protein 5 (Gcn5) as the first histone acetyltransferase (HAT) linked to transcription from studies in Tetrahymena and yeast led to the discovery of the Spt-Ada-Gcn5 acetyltransferase (SAGA) complex [1–3]. SAGA is a multi-module assembly that is best known as a coactivator for gene transcription, and it functions in transcriptional initiation and elongation through enzymatic activities for acetylation and deubiquitylation, ability to bind specific histone modifications, and direct interactions with transcription factors (TFs) such as TATA-binding protein (TBP) [3–6]. GCN5 is the lysine acetyltransferase (KAT) within SAGA, targeting lysines in histones H3 and H2B, and is particularly required for acetylation of histone H3 at lysine 9 (H3K9ac) in mammalian cells [3, 7]. Ubiquitin protease 8 (Ubp8)/ubiquitin-specific peptidase 22 (USP22) is the deubiquitylase (DUB) subunit responsible for catalyzing removal of ubiquitin from monoubiquitinated H2A and H2B [8, 9]. Both GCN5 and Ubp8/USP22 enzymes also target non-histone substrates in and out of the nucleus [10, 11]. GCN5 and adjacent subunit SAGA complex associated factor 29 (SGF29) bind acetylated lysine residues on H3/H4 tails and di/tri-methylation on H3K4 (H3K4me2/3), respectively [12, 13]. Additionally, SAGA components are implicated in non-transcriptional events including maintenance of genome integrity (e.g., GCN5 and USP22), mRNA surveillance (e.g., Sgf73) and export (e.g., Sus1) [14–16].

Yeast Gcn5 and its interacting partner, transcriptional adaptor 2 (Ada2), were found to be part of two complexes designated ADA and SAGA, with respective molecular masses of 0.8 and 1.8 megadalton [3]. Affinity purification and small-scale immunoprecipitation (IP) experiments have demonstrated the conserved existence and composition of SAGA in Arabidopsis, Drosophila and human cells [17–20]. In this review, we highlight differences in complex composition, co-expression patterns, and significant functions of SAGA across all three kingdoms to better understand the conservation and diversity of this essential complex.

## SAGA composition across kingdoms

### Complex modules

SAGA consists of multiple modules: HAT (renamed KAT to reflect its activity towards lysines in non-histone substrates) [21], DUB, TF-binding and core structural modules (Additional file 1: Table S1). Whereas the above modules are found in all eukaryotes, currently an additional splicing module containing splicing factor 3B subunit 3 (SF3B3) and SF3B5 is evident only in metazoans. The core module docks TBP to SAGA via suppressor of ty 3 (Spt3) and Spt8 subunits, which recruits TBP to gene promoters for the formation of the pre-initiation complex (PIC) [22, 23]. A feature of SAGA is that many components and modules are shared with other complexes. In the case of human SAGA, the KAT module is also present in the ADA2A-containing (ATAC) complex, ataxin 7-like 3 (ATXN7L3) and enhancer of yellow 2 (ENY2) of the DUB module interact with other USPs (USP27 and USP51x) to form other DUB complexes, core TATA-binding protein associated factor (TAF) proteins are shared with the TFIID complex, transformation/transcription domain associated protein (TRRAP) is also a subunit of the TAT-interactive protein 60-kDa (TIP60) complex, and the splicing module is common between SAGA and SF3B complexes [24, 25].

The KAT modules of ATAC and SAGA differ in that they contain ADA2A and ADA2B, respectively [26]. Paralogous *ADA2A* and *ADA2B* genes are encoded solely in higher eukaryotic genomes, and whether the GCN5-containing KAT module is incorporated into ATAC or SAGA is determined by its assembly with an either ADA2A or ADA2B protein [27]. In Drosophila, the KAT subunits separately form the ADA complex, while the yeast ADA complex contains two additional subunits, ADA HAT complex component 1 (Ahc1) and Ahc2 [28, 29]. Results of chromatin IP (ChIP) or affinity purification in humans, Drosophila and Arabidopsis, along with the dissociation of DUB module subunits from SAGA by the proteasome regulatory particle demonstrated in yeast, have all implied that the DUB module may also exist independently of SAGA [30–32]. These findings, together with its inherently large composition, suggest that distinct versions of SAGA may be present to execute diverse functions.

### Components important for complex structure

Biochemical and genetic experiments have demonstrated that components of the core structural module, but not of KAT nor DUB modules, are required for the overall structural integrity of SAGA [3, 4]. Deletion of yeast KAT or DUB module subunits only interfered with the assembly of the corresponding module. Recent structural studies by high resolution cryo-electron microscopy revealed that the connections of the core module to the two enzymatic modules are considerably flexible in both yeast and human SAGA complexes [22, 23, 33]. The carboxyl-terminal part of Sgf73 is embedded in the core module, thereby tethering the DUB module to the remainder of SAGA. Interestingly, Arabidopsis lacks an orthologue of Sgf73/ATXN7, and how the plant DUB module interacts with other parts of SAGA is currently unclear [20]. The KAT module has no parts embedded in the core structure and binds to the core surface via Ada3 [22].

### Variations in composition

**Fig. 1:**
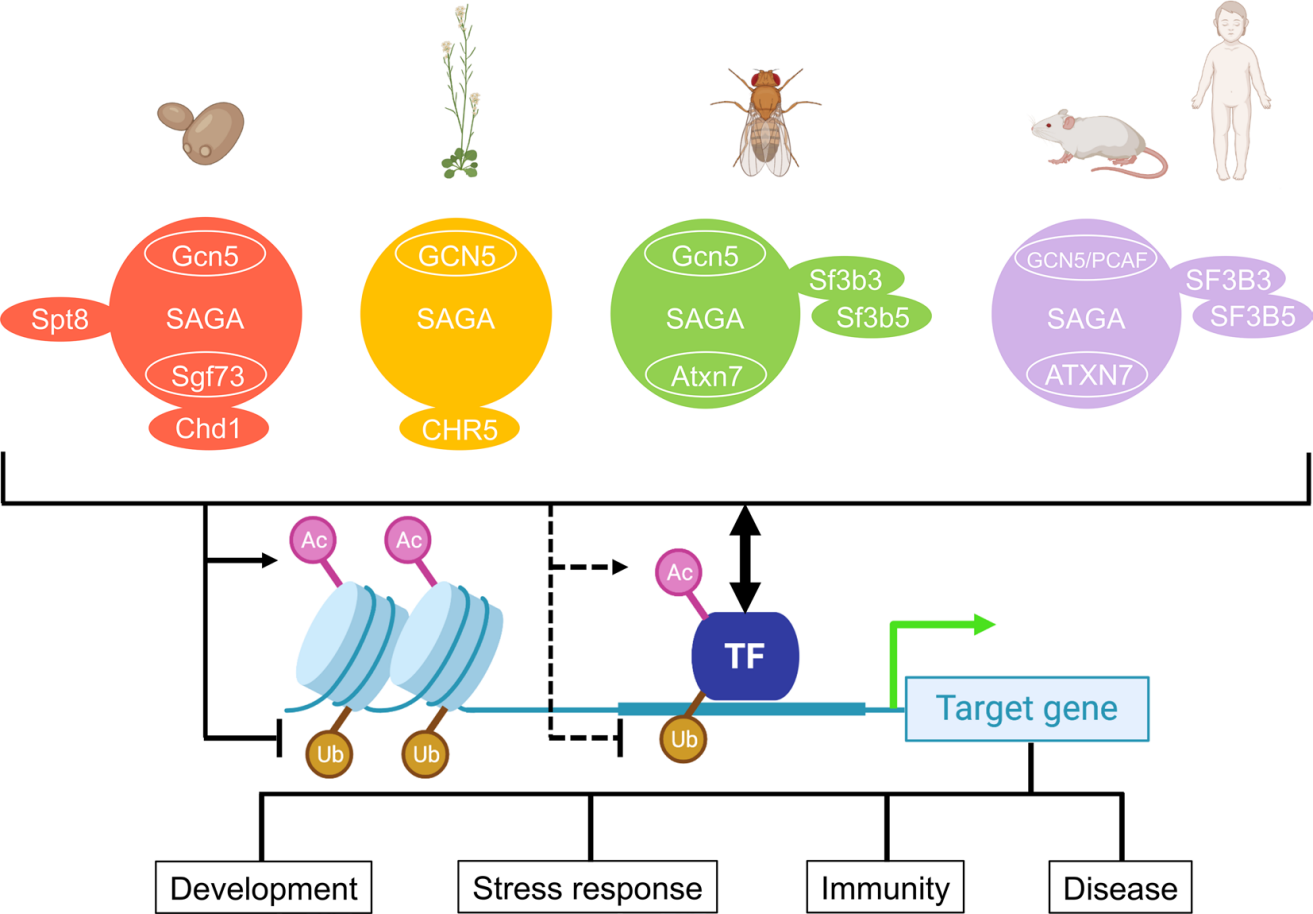
Schematic illustration of SAGA composition variations and function across kingdoms. Orthologues of yeast Spt8 is not found in higher eukaryotic SAGA complexes. Corresponding components of yeast Chd1/Arabidopsis CHR5 are not present in metazoans. Arabidopsis lack orthologues of yeast Sgf73/metazoan ATXN7. SF3B3 and SF3B5 components of the splicing module are not observed in yeast and not confirmed in Arabidopsis. PCAF can replace GCN5 specifically in vertebrates. In all eukaryotes, SAGA catalyzes acetylation (Ac) and removal of ubiquitylation (Ub) on histone tails and interacts with transcription factors (TFs), activating transcription of target genes to further regulate biological outcomes in development, stress response, immunity and disease. SAGA can also acetylate and deubiquitylate TFs to affect their stability and activity

SAGA composition has remained largely conserved throughout eukaryotic evolution, however duplicated homologues that increase structural and functional complexity have been found in higher eukaryotes. Arabidopsis has more duplicates of SAGA components compared to yeast and metazoans, namely TRA1A/B, histone acetyltransferase of the TAFII250 family 1/2 (HAF1/2), TAF6/TAF6B, TAF12/TAF12B, ADA1A/B and SGF29A/B (Additional file 1: Table S1). Plant genomes evolve at higher rates and gene duplication events occur more often in plants than in most other eukaryotes [34]. As a result, a duplicate copy is found for the majority of annotated genes in plant genomes. Affinity purification experiments found that in the cases of TRA1A/B, TAF6/TAF6B, TAF12/TAF12B, ADA1A/B and SGF29A/B, both paralogues interacted with other SAGA subunits [20]. It is currently undetermined whether these paralogues are functionally commutable, or whether specific combinations have unique functions. Specifically in vertebrates, GCN5 has a paralogue named p300/CBP-associated factor (PCAF; Fig. 1 and Additional file 1: Table S1). GCN5 and PCAF are mutually exclusive in the KAT module, resulting in the formation of discrete versions of SAGA as well as ATAC complexes [17, 26].

Orthologues of yeast Spt8 have not been identified in higher eukaryotic SAGA complexes (Fig. 1 and Additional file 1: Table S1). Considering that yeast SAGA-like (SLIK), alternatively called SAGA-altered Spt8-absent (SALSA), complex also lacks Spt8, multicellular SAGA complexes may be more similar to SLIK/SALSA [35, 36]. The SLIK/SALSA complex contains a carboxy-terminally truncated form of Spt7, resulting in the loss of the Spt8-interacting region [37]. However human SPT7 is homologous to the carboxy-terminal part of yeast Spt7, lacks a classical histone fold and bromodomain observed in its yeast orthologue, and instead harbors a bromo-associated domain that resembles a histone-like fold [38]. A component corresponding to yeast chromodomain-containing protein 1 (Chd1), an ATP-dependent chromatin remodeler, has solely been identified in Arabidopsis (CHD3; Fig. 1 and Additional file 1: Table S1) [20, 39]. Although orthologous CHD proteins exist in humans, none were detected to closely interact with SAGA members. As mentioned earlier, plant genomes do not appear to encode a Sgf73/ATXN7 orthologue found in yeast and metazoans. In metazoans, pre-mRNA splicing factors SF3B3 and SF3B5 were identified as SAGA subunits, but their functions in this complex remain elusive [40]. Arabidopsis SF3B3/SIN3A-associated protein 130 (SAP130) protein (encoded by *SAP130A* and *SAP130B* genes) robustly co-purified with canonical SAGA components including ADA2B, SPT3 and TAF10 [20], suggesting that it may also be part of the SAGA complex.

## Expression of SAGA component genes in tissues

**Fig. 2:**
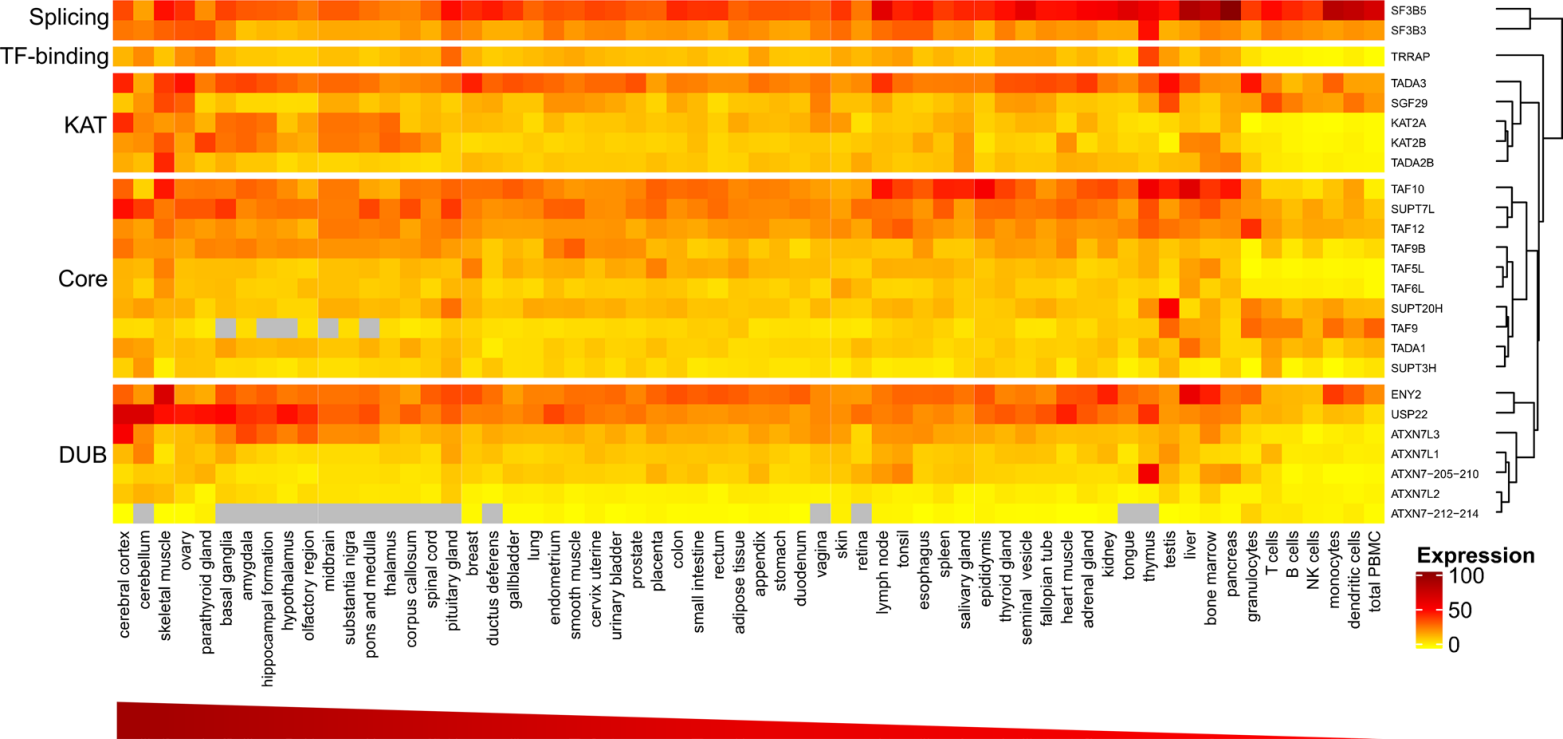
Expression pattern of human SAGA components in organs, tissues and blood cells. Normalized expression (NX) levels were obtained from the consensus dataset on the Human Protein Atlas database. Clustering of expression was generated using the heatmap package in R program. Expression levels are overall high to low from left to right and top to bottom in each module segment. Gray boxes indicate unavailable values. ATXN7-205–210 and ATXN7-212–214 represent two expression profiles of ATXN7 variants

Comprehensive expression studies of genes encoding SAGA components (hereafter referred to as SAGA component genes) in human tissues have not been reported, thus we generated a co-expression heatmap using the consensus dataset from the Human Protein Atlas (HPA), which combines HPA, GTEx and FANTOM5 datasets and includes RNA-sequencing data from 62 tissues and cell types (Fig. 2) [41–43]. The expression profiles show human SAGA component genes are generally ubiquitously expressed. In a majority of tissues, most SAGA component genes are expressed at comparable levels to the average expression of *TBP* (approximately 16 Normalized Expression). Interestingly, a pattern of enhanced expression in brain tissues such as the cerebral cortex and cerebellum, the skeletal muscle and reproductive organs is observed for many SAGA component genes. The high expression of these genes in the cerebellum corresponds to the association of SAGA with a neurodegenerative disease, spinocerebellar ataxia type 7 (SCA7; further discussed in the following section). Catalytic subunit genes *GCN5*, *PCAF* and *USP22* particularly show increased expression in brain tissues. In fact, the top five tissues for *GCN5* expression are all those of the encephalon. Thalamus and amygdala are among the top five tissues for expression of both *GCN5* and *PCAF*. Correspondingly, GCN5, PCAF, as well as other SAGA members have critical developmental functions in the central nervous system (further discussed in the following section) [44, 45].

*PCAF*, *ADA3*, *SGF29*, *ADA2B*, *ENY2*, *USP22*, *TAF10* and *SF3B5* are all highly expressed in the skeletal muscle (Fig. 2), suggesting that the PCAF-containing version of SAGA may have a larger role in this tissue. Several studies have indicated the direct involvement of PCAF in muscle differentiation via disparate molecular pathways [46–48]. The KAT activity of PCAF on histones and DNA-binding myoblast determination protein (MyoD) activates the myogenic program, presumably in a multimeric complex that comprised PCAF, E1A binding protein p300/CREB-binding protein (p300/CBP) and MyoD [46, 47]. In committed muscle cell precursors, PCAF promotes histone deacetylase 2 (HDAC2) acetylation and recruitment to lamin A/C at the nuclear lamina, displacing HDAC2 from MyoD, thus preventing deacetylation and inactivation of MyoD at the promoters of myogenic loci [48]. Developmental phenotypes were not observed in *Pcaf* null mice [49], suggesting redundancy between GCN5 and PCAF in muscle tissues.

*SF3B5* is expressed at extremely high levels, notably in pancreas, liver, bone marrow and blood cells (Fig. 2), which may reflect its functions in both SAGA and non-SAGA complexes. Interestingly, the expression of *ATXN7* splice variants are also abundant in the bone marrow and blood cells and can be grouped into two profiles, with variants 205, 207, 209, 210 significantly enriched in the thymus, other lymphoid tissues and bone marrow, and variants 212 to 214 more distributed in blood cells. Increased expression in blood cells, including T cells and others, is also observed for *SGF29*, *SPT20*, *TAF9* and *ADA1*. In agreement with these expression patterns, SGF29, SPT20, ADA1 and other SAGA components were identified as positive modulators of forkhead box P3 (FOXP3) expression in regulatory T cells (Tregs; further discussed in the following section) [50, 51].

Enrichment of SAGA component transcripts in reproductive tissues is conserved among higher eukaryotes. Additionally, numerous component genes are abundantly expressed in Arabidopsis and Drosophila embryos, but expression data in mammalian embryos are lacking [52–54]. In Arabidopsis, SAGA component genes show an increased expression pattern in the embryo, endosperm and maternal seed coat that support the growth and development of the embryo [52]. Accordingly, TAF13 was shown to be essential for embryogenesis, possibly via interaction with Medea (MEA) and Swinger (SWN), two subunits of the plant polycomb repressive complex 2 (PRC2) [55]. The *taf13* mutant displays embryo arrest at the 8–16 cell stage and over-proliferation in the endosperm, resembling phenotypes of plants with PRC2 mutations. In Drosophila, expression of nearly all SAGA component genes, including *Gcn5*, *Usp22/Nonstop*, *Ada1*, *Ada2b*, *Ada3*, *Sgf11*, *Sgf29*, *Spt3*, *Spt7*, *Spt20*, *Taf5/Wda*, *Taf6/Saf6* and *Taf9/E(y)1*, is enriched in the ovary and during the earliest stages of embryogenesis [53, 54]. In humans, SAGA component genes have a pattern of upregulated expression in the ovary, testis and breast of the reproductive system (Fig. 2). These expression data are consistent with the reported pivotal function of GCN5 in mammalian spermiogenesis and fruitfly oogenesis [56, 57]. Mice deficient of *Gcn5* show aberrant chromatin dynamics and increased nucleosome retention in the sperm, ultimately leading to male fertility defects [57]. Taken together, the enhanced expression of SAGA component genes in reproductive tissues of higher eukaryotes is accordant with their requirement for successful reproduction.

## Prominent roles of SAGA across kingdoms

### Development

SAGA KAT and DUB module components that are essential for growth and development in metazoans, of which mutations cause lethality, are contrarily often not essential in yeast and plants (Additional file 1: Table S1). Differences in essentiality of homologous genes may reflect specific SAGA functions in particular metazoan or mammalian tissues or processes, such as the requirement of USP22 for the formation of the mouse placenta [58]. At the molecular level, essentiality of genes may be altered by sequence changes that could potentially cause conformational differences in the encoded proteins, or the emergence of redundant genes. For instance, a large N-terminal extension, named the PCAF domain, exclusively exists in the metazoan GCN5 and is important for nucleosome binding [59]. Albeit non-essential, deletion of yeast *GCN5* causes pleiotropic phenotypes including defective cell cycle progression and shortened replicative lifespan [60, 61]. Hypomorphic mutants of Arabidopsis *GCN5* also display pleiotropic defects such as dwarfism and aberrant root, leaf and floral development, but a null mutant has yet to be reported [62]. In Drosophila, *Gcn5* hypomorphic alleles cause abnormal appendages and absence of abdominal cuticle deposition, while null alleles hamper onset of oogenesis and metamorphosis [56]. *Gcn5* null mice die soon after gastrulation, reaching approximately one-third of embryo development [49]. Deletion of PCAF, which is 71% identical to GCN5, in mice does not result in overt developmental defects before or after birth. However, embryos deficient for both *Gcn5* and *Pcaf* die earlier than those null for *Gcn5*, indicating partial redundancy between these KATs during early embryogenesis.

Tra1 is essential in budding yeast (*Saccharomyces cerevisiae*) but not in fission yeast (*Schizosaccharomyces pombe*), while deletions of the genes encoding the Taf proteins, which also function in the TFIID complex, cause lethality in both yeast species [63–65]. Depletion of other SAGA core components that are crucial for structural integrity, including Spt7, Spt20 and Ada1, results in severe growth phenotypes, whereas mutations in components not critical for complex integrity, including Gcn5, Ada2, Ada3, Spt3 and Spt8, display moderately slow growth under various nutritional and stress conditions [4]. Deletion of *UBP8* has a positive effect for extending lifespan of yeast cells [66]. In contrast to yeast as was the case for GCN5, both fruitfly and mouse ADA3, TRRAP/Tra1, ATXN7L3/Sgf11 and USP22/Nonstop are essential for early development [67–73]. Lethality of *Trrap* or *Ada3* null mutations in mice is linked to cell cycle progression defects [67, 72]. Death of mouse *Usp22* mutants is due at least in part to failure in placenta formation, which is related to inhibition of transforming growth factor beta (TGF-β) and receptor tyrosine kinase (RTK) signaling pathways that control vascularization [58].

Both SAGA-specific Ada2b and ATAC-specific Ada2a are essential in Drosophila, but at different developmental phases; *Ada2a* and *Ada2b* null mutants are larva and pupa lethal, respectively, indicating loss of *Ada2a* is slightly more severe than loss of *Ada2b* [74]. Arabidopsis *ada2b* null mutants exhibit pleiotropic phenotypes mirroring *gcn5* mutants but are not lethal, whereas *ada2a* null mutants do not exhibit pronounced phenotypes [62]. Of note, sequence identities indicate that Arabidopsis ADA2A is more closely related to human ADA2B while Arabidopsis ADA2B is more similar to human ADA2A. ADA2A and ADA2B share 45% sequence identity in Arabidopsis, 17% in Drosophila, and 27% in humans. In accordance with their functions in different complexes, the two paralogues are predictably non-redundant, as Ada2a could not rescue *Ada2b* mutant flies, and vice versa [75]. Rescue experiments using chimeric proteins indicate the C-terminal region determines the incorporation into SAGA or ATAC complexes. The N-terminus of Ada2b fused with C-terminus of Ada2a could partially rescue *Ada2a* mutants and restore ATAC-mediated H4K5ac and H4K12ac levels; the N-terminus of Ada2a fused with C-terminus of Ada2b could fully rescue *Ada2b* mutants and restore SAGA-targeted H3K9ac and H3K14ac levels. Related to this finding, a SWI3-RSC8-MOIRA (SWIRM) domain lies within the C-terminus of Ada2a but appeared absent in Drosophila Ada2b. The SWIRM domain is annotated in both of the human paralogues, therefore it remains to be ascertained whether the C-terminal region consistently determines complex-specific incorporation in humans.

### Stress response

Yeast SAGA members contribute to activation of various sets of stress-regulated genes under conditions such as glucose, osmotic and ER stresses [76–78]. For instance, SAGA is required for the recruitment of Mediator and switch/sucrose non-fermentable (SWI/SNF) complexes to promoters of genes activated by low glucose, as demonstrated by comparison of ChIP patterns in wild type and *gcn5* or *ada1* deletion strains [78]. Deletion of *GCN5*, *ADA2*, *ADA3*, or *SPT20* results in partially or completely defective unfolded protein response following ER stress due to failure of transcriptional induction of response programs [76]. Concordantly, human SAGA is recruited to ER stress-regulated genes possibly via interaction between GCN5 and the nuclear factor Y (NFY) that binds to ER stress-response element sequences in promoters [30].

In Arabidopsis, GCN5 and ADA2B have been widely studied in abiotic stress response [79]. Under heat stress, GCN5 is enriched at promoters of genes encoding heat stress TFs, heat shock factor A3 (HSFA3) and UV hypersensitive 6 (UVH6), where it promotes H3K9ac and H3K14ac that are associated with activation of these genes, thus preserving thermotolerance [80]. Upon cold exposure, GCN5, ADA2B and C-repeat binding factor 1 (CBF1) activate cold-responsive genes and consequently increase freezing tolerance [62]. GCN5 and ADA2B also mediate H3K9ac and H3K14ac to facilitate expression of salt stress-responsive genes, including genes required for maintenance of cell wall integrity and salt tolerance [81, 82]. The involvement of SAGA components in drought response was manifested in the black cottonwood tree *Populus trichocarpa* [83]. GCN5 and ADA2B promote H3K9ac, RNA polymerase II enrichment, and expression of drought-responsive genes upon recruitment by a drought-induced TF, abscisic acid-responsive element binding protein 1 (AREB1). Collectively, GCN5 and ADA2B regulate plant adaptation to abiotic stresses by interaction with stress-induced TFs and transcriptional activation of stress-responsive genes.

SAGA functions in stress response have been less characterized in metazoans, however they are repeatedly linked to disease states (discussed below). Commonly in mammals, physiological stress and disorders are closely associated. One example is the acetylation and inactivation of peroxisome proliferators gamma coactivator 1 (PGC-1) family of transcriptional coactivators including PGC-1α and PGC-1β, which are conferred by GCN5 and mediate glucose metabolism [84, 85]. Dysregulation of these pathways generates metabolic stress and further contributes to pathogenesis and disease progression.

### Immunity

SAGA components regulate the development of specific populations of immune cells. During granulocyte differentiation, GCN5 acts as a negative regulator through catalyzing acetylation of a TF, CCAAT enhancer binding protein alpha (C/EBPα), thereby inhibiting its DNA-binding ability [86]. On the other hand, GCN5 is required for T cell differentiation and full activation in immune response, wherein nuclear factor of activated T cells (NFAT) recruits GCN5 to catalyze H3K9ac at the *interleukin-2* (*IL-2*) promoter and to activate *IL-2* transcription upon antigen stimulation [87]. Acetylation of early response growth protein 2 (EGR2) by GCN5 activates the TF activity of EGR2 and stimulates the maturation of invariant natural killer T cells [88]. PCAF plays a larger role in facilitating production of induced Tregs than GCN5, which is achieved by PCAF-mediated IL-2 generation and mothers against decapentaplegic homologue 3 (SMAD3) phosphorylation in TGF-β signaling [89]. Double knockout of *Gcn5* and *Pcaf* in mice Tregs decreases Treg stability in peripheral lymphoid tissues and subsequently causes lethal autoimmunity. Recently, CRISPR screens performed by two groups simultaneously identified SAGA components, including USP22, ATXN7L3, ADA1, ADA2B, ADA3, SGF29, SPT20, TAF5L and TAF6L, as novel regulators promoting expression of FOXP3, a master TF governing the stability and suppressor activity of Tregs [50, 51]. USP22 maintains FOXP3 levels through both transcriptional and post-translational regulations: USP22 reduces H2B ubiquitination at the *FOXP3* locus and concurrently mediates deubiquitination of FOXP3 protein, thereby sustaining transcription and preventing protein degradation. Finally, in addition to direct regulation of immune cells, GCN5 and PCAF suppress TANK-binding kinase 1 (TBK1) activity in the cytoplasm by blocking phosphorylation events on TBK1, ultimately inhibiting innate immune signaling and interferon production upon viral infection [90]. Altogether, these studies demonstrate that SAGA components are critical for innate as well as adaptive immunity and are potential therapeutic targets in the treatment of immune disorders such as autoimmune diseases.

### Neural development and neurodegenerative diseases

SAGA members are involved in neural development of the central and peripheral nervous systems, and aberrations in a single component could lead to detrimental consequences. Mice with *Gcn5*-deficient neural stem and precursor cells have a microcephaly phenotype caused by reduction of brain mass, whereas mice homozygous for a catalytic-dead allele of *Gcn5* are defective in cranial neural tube closure, resulting in exencephaly [44, 91]. Although *Pcaf* null mice do not display developmental defects, they exhibit impaired memory and learning ability, as well as an overreactive emotional response to acute stress [45]. Knockdown of *Usp22* facilitates neural differentiation in the developing mouse brain [92]. USP22 is the predominant DUB that deubiquitinates and stabilizes hairy and enhancer of split 1 (HES1), a transcriptional repressor that maintains the undifferentiated state of neural stem cells. Drosophila Usp22/Nonstop and Sgf11 regulate neural development in the visual system via H2B deubiquitination, however loss of their individual functions has no effects on global H3K9Ac [70]. KAT module mutants *Gcn5* and *Ada2b* display similar defects in axon targeting as those observed in *Nonstop* and *Sgf11* mutants, but are coupled with a clear reduction of global H3K9Ac levels. The flies bearing mutations interfering with DUB or KAT activities show overlapping and distinct transcriptional profiles, indicating the two enzymatic modules have both common and discrete targets.

Expansion of a polyglutamine tract in ATXN7 (PolyQ-ATXN7) results in SCA7, a disease that is characterized by cerebellar and retinal degeneration. PolyQ-ATXN7 can incorporate into the DUB module of SAGA, not altering DUB activity but forming a dominant-negative version of SAGA with inhibited coactivator activity [93, 94]. PolyQ-ATXN7 dramatically attenuates expression of rod-specific genes such as *rhodopsin* (*Rho*) and *G protein subunit alpha transducin 1* (*Gnat1*), leading to progressive loss of photoreceptor function in the SCA7 mouse model [95]. Deletion of *Gcn5* in SCA7 mice accelerates retinal degeneration and onset of ataxia, further suggesting a crucial role for SAGA in SCA7 pathogenesis [96].

### Cancer

Genome-wide CRISPR-based screens have identified human SAGA component genes as dependencies for viability of different cancer cell types. GCN5 was isolated to be essential for acute myeloid leukemia (AML) cell proliferation; ADA2B and several other SAGA components were identified as selective dependencies essential for the growth and survival of *MYCN*-amplified neuroblastoma cell lines [97, 98]. The large-scale Cancer Dependency Map (DepMap) [99, 100] revealed SAGA components are important for viability of a wide spectrum of cancer cell lines, including those of multiple myeloma, lung and breast cancer. Dependency scores of SAGA component genes exhibit a strong positive correlation with each other, indicating this group of genes function together as one regulatory complex. Ectopic expression of SAGA components can promote oncogenesis or tumor progression. GCN5 acetylates and reinforces stability of the oncogenic MYC TF that is overexpressed in a majority of human cancers, and in turn MYC facilitates expression of SAGA component genes in a positive feedback loop [101, 102]. Moreover, MYC directly interacts with GCN5 and TRRAP to recruit SAGA to its downstream targets for transcriptional activation [103]. GCN5 expression and function therefore accelerates growth of MYC-overexpressing AML, Burkitt’s lymphoma (BL), hepatocellular carcinoma (HCC), colon and non-small cell lung cancer (NSCLC) in cellular systems and, shown in BL, HCC and NSCLC, in vivo mouse models [97, 104–109]. Overexpression of ADA3 enhances proliferation of breast cancer cells, which again is associated with MYC upregulation [110]. A microarray screen identified *USP22* as part of the 11-gene “death from cancer” signature driven by B cell-specific Moloney murine leukemia virus integration site 1 (BMI-1), a PRC1 member, in metastatic prostate tumors [111]. This BMI-1 pathway signature predicts disease recurrence, distant metastasis, and poor prognosis in 11 types of malignancies.

In addition to MYC, SAGA components also regulate cancer development through post-translational modifications of other non-histone substrates such as chromatin modifiers. PCAF acetylates enhancer of zeste homologue 2 (EZH2), a histone methyltransferase and PRC2 subunit, augmenting silencing of tumor suppressor genes and resulting in progression of lung adenocarcinoma [112]. Acetylation of intestine‐specific homeobox (ISX) and bromodomain‐containing protein 4 (BRD4) by PCAF promotes physical association and nuclear translocation of these proteins to drive epithelial–mesenchymal transition (EMT) through activation of EMT genes in lung cancer cells [113]. In prostate cancer, USP22 mediates the deubiquitination of xeroderma pigmentosum complementation group C (XPC), a nucleotide excision repair protein that detects DNA damage [114].

## Conclusions and future perspectives

Unlike other GCN5-containing complexes such as the metazoan-specific ATAC complex, SAGA is uniquely conserved in all eukaryotes. The recently elucidated high-resolution structures of SAGA from two yeast genera and from humans enlightened our understanding of its dynamic conformation in unicellular and higher eukaryotes [22, 23, 33]. Attributable to its intrinsic large composition and structural flexibility of the KAT and DUB modules in relation to the core module, each module may have independent roles in the process of transcriptional regulation, which is consistent with different impacts to gene expression profiles upon mutation of components belonging to separate modules [70]. Dissimilar mutant phenotypes also reflect different individual roles of subunits, such as cofactors in the enzymatic modules and core subunits involved in maintenance of complex integrity that are dispensable or absolutely essential [4]. In many cases, SAGA components also perform functions in other protein complexes, complicating interpretation of genetic phenotypes.

Our comparison of the compositions, co-expression patterns, and defined functions for SAGA suggests the existence of alternative versions of the complex across kingdoms as well as within a species (Figs. 1, 2). Some studies indicated that SAGA is a general coactivator required broadly for transcription [6, 115], whereas others conversely elucidated its specific targeting to gene promoters through interactions with particular transcription factors [30, 69, 100, 116]. The hypothesis that SAGA has tissue, developmental, physiological and pathological state-dependent compositions, interactions and functions would explain and support both findings. Although it is established that SAGA performs critical functions in development and stress response conservatively across Fungi, Plantae and Animalia kingdoms, and in immunity and disease in metazoans (Fig. 1), the underlying mechanisms of how distinct compositions assemble and functions are executed remain to be addressed. Our co-expression analysis of transcript levels indicates tissue-specific expression may provide one layer of regulation for SAGA composition, but protein quantification across tissues for all SAGA components is required to further validate this possibility. Comprehensive definition of SAGA interaction partners from different cell types and conditions would also generate important insights as to how SAGA is recruited to its targets in varying biological contexts. Employment of recent techniques including Assay for Transposase-Accessible Chromatin followed by sequencing (ATAC-seq) could aid in complementing the challenges of performing ChIP for certain SAGA members and greatly advance our knowledge of the impact of SAGA on chromatin organization and accessibility.

The discovery of Gcn5 as a gene-activating KAT more than two decades ago revolutionized our understanding of the connection between histone modification and transcriptional regulation [2]. However, we are still only at the tip of the iceberg in exploring the potential of manipulating activities of GCN5 and other SAGA components to combat human ailments. In addition to directly impact tumor formation and progression, for example, targeting of SAGA components could affect response to immunotherapy given that SAGA functions in both innate and adaptive immune systems. Indeed, loss of PCAF in mice decreases lung adenocarcinoma tumor volume and augments anti-tumor immunity without triggering autoimmune side effects, potentiating clinical benefits from PCAF inhibition in combination with immunotherapy [89]. Data mining of publicly available resources unveiled the importance of poorly characterized SAGA component genes for viability of cancer cells (99, 100), thus clarifying these component functions may uncover new therapeutic targets for cancer treatment. Finally, the conserved role of SAGA in neural development among metazoans and its understudied role in muscle development or maintenance prompt further investigation of SAGA-mediated mechanisms in neurodegenerative and neuromuscular diseases. These research directions offer exciting prospects for future definition of SAGA functions in specific contexts.

## Supplementary Information

**Additional file 1: Table S1.** SAGA composition and essentiality of each component in yeast, Arabidopsis, Drosophila and mice. *Deletion of TRA1 is lethal in *S. cerevisiae* but viable in *S. pombe*. **Homozygotes die at 7 to 8 weeks. ND, not determined.

## Data Availability

Not applicable.

## Abbreviations

Gcn5: General control nonrepressed protein 5
HAT: Histone acetyltransferase
SAGA complex: Spt-Ada-Gcn5 acetyltransferase complex
TF: Transcription factor
TBP: TATA-binding protein
KAT: Lysine acetyltransferase
H3K9/14ac: Acetylation at lysine 9/14 in histone H3
Ubp8: Ubiquitin protease 8
USP22: Ubiquitin-specific peptidase 22
H3K4me2/3: Di/tri-methylation at lysine 4 in histone H3
Ada: Transcriptional adaptor
IP: Immunoprecipitation
SF3B3/5: Splicing factor 3B subunit 3/5
Spt: Suppressor of ty
PIC: Pre-initiation complex
ATAC complex: ADA2A-containing complex
ATXN7(L3): Ataxin 7(-like 3)
ENY2: Enhancer of yellow 2
TAF: TATA-binding protein associated factor
TRRAP: Transformation/transcription domain associated protein
TIP60: TAT-interactive protein 60-kDa
Ahc1/2: ADA HAT complex component 1/2
ChIP: Chromatin IP
HAF: Histone acetyltransferase of the TAFII250 family
PCAF: P300/CBP-associated factor
SLIK complex: SAGA-like complex
SALSA complex: SAGA-altered SPT8-absent complex
Chd: Chromodomain-containing protein
HPA: Human Protein Atlas
MyoD: Myoblast determination protein
p300/CBP: E1A binding protein p300/CREB-binding protein
HDAC2: Histone deacetylase 2
FOXP3: Forkhead box P3
Treg: Regulatory T cell
MEA: Medea
SWN: Swinger
PRC1/2: Polycomb repressive complex 1/2
TGF-β: Transforming growth factor beta
RTK: Receptor tyrosine kinase
SWIRM: SWI3-RSC8-MOIRA
SWI/SNF complex: Switch/sucrose non-fermentable complex
NFY: Nuclear factor Y
HSFA3: Heat shock factor A3
UVH6: UV hypersensitive 6
CBF1: C-Repeat binding factor 1
AREB1: Abscisic acid-responsive element binding protein 1
PGC-1: Peroxisome proliferators gamma coactivator 1
C/EBPα: CCAAT Enhancer binding protein alpha
NFAT: Nuclear factor of activated T cells
IL-2: Interleukin-2
EGR2: Early response growth protein 2
SMAD3: Mothers against decapentaplegic homologue 3
TBK1: TANK-binding kinase 1
HES1: Hairy and enhancer of split 1
PolyQ-ATXN7: Polyglutamine expanded ATXN7
SCA7: Spinocerebellar ataxia type 7
Rho: Rhodopsin
Gnat1: G protein subunit alpha transducin 1
AML: Acute myeloid leukemia
BL: Burkitt’s lymphoma
HCC: Hepatocellular carcinoma colon cancer
NSCLC: Non-small cell lung cancer
BMI-1: B cell-specific Moloney murine leukemia virus integration site 1
EZH2: Enhancer of zeste homologue 2
ISX: Intestine‐specific homeobox
BRD4: Bromodomain‐containing protein 4
EMT: Epithelial–mesenchymal transition
XPC: Xeroderma pigmentosum complementation group C
