# Supplementary material for: Conservation and diversity of the eukaryotic SAGA coactivator complex across kingdoms
Source: Epigenetics Chromatin. 2021 Jun 10;14:26. doi: 10.1186/s13072-021-00402-x (PMC8194025; doi:10.1186/s13072-021-00402-x)
Supplement: Supplementary file 1 — Additional file 1: Table S1. SAGA composition and essentiality of each component in yeast, Arabidopsis, Drosophila and mice. *Deletion of TRA1 is lethal in S. cerevisiae but viable in S. pombe. **Homozygotes die at 7 to 8 weeks. ND, not determined. [file 13072_2021_402_MOESM1_ESM.pdf]

Table 1

Module	Yeast Subunit	Yeast Essentiality	Arabidopsis Subunit	Arabidopsis Essentiality	Fruitfly Subunit	Fruitfly Essentiality	Human/Mouse Subunit	Human/Mouse Essentiality
TF-binding	Tra1	Essential/Non-essential*	TRA1A<br>TRA1B	ND<br>ND	Tra1 (Nipped-A)	Essential	TRRAP	Essential
Core	Spt3	Non-essential	SPT3 (TAF13)	Essential	Spt3 (CG3169)	ND	SPT3 (SUPT3; SUPT3H)	Essential
	Spt7	Non-essential	SPT7 (HAF1)	Essential	Spt7 (CG6506)	ND	SPT7 (SUPT7L; STAF65G)	ND
	Spt8	Non-essential						
	Spt20 (Ada5)	Non-essential	SPT20 (PHL)	Non-essential	Spt20 (CG17689)	ND	SPT20 (SUPT20H; P38IP; FAM48A)	ND
	Taf5	Essential	TAF5	Essential	Taf5 (Wda)	Essential	TAF5L	ND
	Taf6	Essential	TAF6<br>TAF6B	Essential<br>ND	Taf6 (Saf6)	ND	TAF6L	ND
	Taf9	Essential	TAF9	ND	Taf9 (E(y)1)	Essential	TAF9<br>TAF9B	ND<br>ND
	Taf10	Essential	TAF10 (STG1)	ND	Taf10B	ND	TAF10	Essential
	Taf12	Essential	TAF12<br>TAF12B	ND<br>Non-essential	Taf12	Essential	TAF12	ND
	Ada1 (Hfi1)	Non-essential	ADA1A<br>ADA1B	ND<br>ND	Ada1-1 (CG31865)<br>Ada1-2 (CG31866)	ND<br>ND	ADA1 (TADA1)	ND
KAT	Gcn5 (Ada4)	Non-essential	GCN5 (HAG1)	ND	Gcn5	Essential	GCN5 (KAT2A)<br>PCAF (KAT2B)	Essential<br>Non-essential
	Ada2	Non-essential	ADA2B	Non-essential	Ada2b	Essential	ADA2B (TADA2B)	ND
	Ada3 (Ngg1)	Non-essential	ADA3	ND	Ada3 (CG7098)	Essential	ADA3 (TADA3)	Essential
	Sgf29	Non-essential	SGF29A<br>SGF29B	Non-essential<br>ND	Sgf29 (CG30390)	ND	SGF29 (CCDC101)	ND
DUB	Ubp8	Non-essential	UBP22	Non-essential	Nonstop	Essential	USP22 (UBP22)	Essential
	Sus1	Non-essential	ENY2	Essential	E(y)2	ND	ENY2	ND
	Sgf11	Non-essential	SGF11	Non-essential	Sgf11	Essential	ATXN7L3	Essential
	Sgf73	Non-essential			Atxn7 (CG9866)	Essential	ATXN7<br>ATXN7L1<br>ATXN7L2	Essential**<br>ND<br>ND
Splicing			SAP130	ND	Sf3b3	Essential	SF3B3 (SAP130)	ND
					Sf3b5	Essential	SF3B5	ND
Other	Chd1	Non-essential	CHD3	Non-essential				
